# Marine Algae as a Potential Source for Anti-Obesity Agents

**DOI:** 10.3390/md14120222

**Published:** 2016-12-07

**Authors:** Chu Wan-Loy, Phang Siew-Moi

**Affiliations:** 1 School of Postgraduate Studies, International Medical University, Kuala Lumpur 57000, Malaysia; wanloy_chu@imu.edu.my; 2 Institute of Biological Sciences, University of Malaya, Kuala Lumpur 50603, Malaysia; 3 Institute of Ocean & Earth Sciences (IOES), University of Malaya, Kuala Lumpur 50603, Malaysia

**Keywords:** obesity, algae, seaweeds, fucoxanthin, alginates, fucoidans, phlorotannins, pancreatic lipase inhibitors

## Abstract

Obesity is a major epidemic that poses a worldwide threat to human health, as it is also associated with metabolic syndrome, type 2 diabetes and cardiovascular disease. Therapeutic intervention through weight loss drugs, accompanied by diet and exercise, is one of the options for the treatment and management of obesity. However, the only approved anti-obesity drug currently available in the market is orlistat, a synthetic inhibitor of pancreatic lipase. Other anti-obesity drugs are still being evaluated at different stages of clinical trials, while some have been withdrawn due to their severe adverse effects. Thus, there is a need to look for new anti-obesity agents, especially from biological sources. Marine algae, especially seaweeds are a promising source of anti-obesity agents. Four major bioactive compounds from seaweeds which have the potential as anti-obesity agents are fucoxanthin, alginates, fucoidans and phlorotannins. The anti-obesity effects of such compounds are due to several mechanisms, which include the inhibition of lipid absorption and metabolism (e.g., fucoxanthin and fucoidans), effect on satiety feeling (e.g., alginates), and inhibition of adipocyte differentiation (e.g., fucoxanthin). Further studies, especially testing bioactive compounds in long-term human trials are required before any new anti-obesity drugs based on algal products can be developed.

## 1. Introduction

Obesity is a metabolic disorder characterized by excess body fat accumulation, with Body Mass Index (BMI) of more than 30 kg/m^2^, reflected by an increased waist circumference [1]. The disease is mainly due to excessive food intake, lack of physical activity and genetic factors. Based on a pooled analysis of BMI from populations of 200 countries, a recent study projected that by 2025, global obesity will surpass 6% in men and 9% in women [2]. Obesity has become a global threat to public health as it impairs quality of life and imposes a huge expenditure on healthcare cost. In obese individuals, there is an increased risk of developing type 2 diabetes, dyslipidemia, hypertension, cardiovascular (CVS) disease, non-alcoholic fatty liver disease (NFLD) and certain types of cancer [3].

Excess fats (triacylglycerols) are stored in adipocytes, which may serve as endocrine cells that secrete biologically active mediators. Such mediators are known as adipokines/chemokines which include leptin, adiponectin, resistin, tumor necrosis factor-α (TNF-α) and monocyte chemoattractant protein-1 (MCP-1). The adipokines may alter insulin sensitivity, glucose and lipid metabolism in muscles, liver and adipose tissues [4]. Obesity also induces a state of low-level inflammation, which is a determinant in the development of complications such as insulin resistance, type 2 diabetes, artherosclerosis and NFLD [5].

Measures to reduce body weight is part of the strategies in obesity treatment. Effective long term weight loss can be succeeded through permanent changes in dietary quality, food intake, and physical activity [6]. Diet quality can be improved by reducing the consumption of energy-dense foods and by increasing the intake of dietary fibers. For individuals with BMI ≥ 30 kg/m^2^, as well as those with BMI of 25.0–29.9 kg/m^2^ plus two or more disease risk factors, lifestyle modification by adhering to a program of diet, exercise, and behavior is the recommended treatment for obesity [7]. Pharmacological interventions in treating obesity are recommended for those with BMI ≥ 30 kg/m^2^ or with a BMI ≥ 27 kg/m^2^ in the presence of two or more obesity-related comorbidities such as coronary heart disease or type 2 diabetes.

There are two main categories of anti-obesity drugs, namely agents that are able to reduce or limit energy absorption, and those that aim to decrease fat mass by increasing energy expenditure or redistributing adipose tissue [8]. Only very few anti-obesity drugs are commercially available although many are in pre-clinical and clinical trials. There are four drugs currently approved by the Food and Drug Administration (FDA) for chronic management of obese adults: orlistat, locaserin, phentermine/topiramate (extended release) and naltrexone/bupropion (extended release) [6]. Of these, orlistat is the sole agent licensed for management of obesity in the UK. There is a huge projected market size for anti-obesity drugs, which is expected to reach US$ 2.4 billion by 2021 [9].

Pancreatic lipase inhibitors are amongst the drugs used for weight control [6]. Inhibition of pancreatic lipase decreases the free fatty acids and monoacylglycerols in the intestinal lumen, resulting in decreased amounts of triacylglycerols to be absorbed. Orlistat is a potent drug of this category that is currently available in the market, widely used for weight management in combination with reduced caloric diet. Other drugs that target lipid absorption include those that inhibit diacylglycerol O-acyltransferase (DGAT), which results in the inhibition of triacylglycerol synthesis in enterocytes. However, such drugs have only been tested on animals and the experimental data may not be generalized to humans [8].

The side-effects from anti-obesity drugs have been a major concern for their therapeutic usage. Among the anti-obesity drugs, sibutramine is the first that has been withdrawn from the market due to the side effects of cardiovascular events and strokes. As such, there is an urgent need for safe anti-obesity drugs that are therapeutically potent [10]. Thus, there has been an increasing interest in the search for natural products, especially phytonutrients with anti-obesity activities. Marine organisms have been regarded as a potential source of bioactive compounds with anti-obesity activities [11]. Most of such compounds are produced by marine algae, especially brown seaweeds [12]. Algal compounds that have been shown to have potential anti-obesity activities include fucoxanthin, phlorotannins, fucoidan and alginates. This review aims to provide an overview of the advances in research related to anti-obesity effects of marine algae, with emphasis on these algal compounds as potential anti-obesity agents

## 2. Seaweeds as Food and Their Potential Anti-Obesity Effects

Seaweeds are important dietary component of people in countries such as Japan and Korea. For instance, seaweed is served in approximately 21% of meals in Japan [13], and the daily consumption per person has been relatively consistent between 1955 (4.3 g/day) and 1995 (5.3 g/day) [14]. The major seaweeds consumed include Wakame (*Undaria*), Nori (*Porphyra*), Konbu (*Laminaria*) and Hijiki (*Hizikia*). The annual global market value of the seaweed industry amounts to US$5.5–6 billion per year and the main uses of seaweeds are for food and phycocolloids [15]. About one million tonnes of seaweeds are used per year for the production of phycolloids, namely agar (US$132 million), alginates (US$13 million) and carrageenans (US$240 million).

Seaweeds are of high nutritious value due to their high contents of fibers, minerals and polyunsaturated fatty acids [16]. In addition, seaweeds contain a variety of bioactive compounds such as phlorotannins and polysaccharides not found in terrestrial plants, which may play a role in modulating chronic diseases [17]. Epidemiological studies have shown that there is an association between dietary intake of seaweeds and a reduced prevalence of chronic diseases including cardiovascular disease, hyperlipidemia and cancer [17]. Bioactive molecules such as polyunsaturated fatty acids and polyphenolic compounds in seaweeds have the potential health benefits of preventing and managing type 2 diabetes [18]. Extracts from brown seaweeds have been shown to inhibit α-glucosidase and dipeptidyl-peptidase-4 (DPP-4) and have the ability to stimulate incretin hormone secretion [19]. There have also been many reports on the potential therapeutic benefits of seaweed consumption in the management of body weight and obesity [12].

Studies on anti-obesity effects of seaweeds were conducted using whole seaweed meal, seaweed extracts or seaweed bioactives, mainly on cell-based and animal models although the number of human trials have been increasing. For example, feeding of seaweed powder of *Sargassum polycystum* suppressed weight gain in rats fed with high fat diet and reduced the plasma levels of cholesterol and triacylglycerols [20]. In another study, feeding of the brown seaweed *Undaria pinnatifida* showed positive effects on body weight gain, energy consumption, and serum levels of glucose and insulin in diet-induced obese mice [21]. In addition, there was decreased expression of the inflammatory marker interleukin-6 (IL-6), increased energy expenditure, and decreased lipogenesis that resulted in more and smaller adipocytes in retriponeal tissue. The authors suggested that the reduction in body weight was not attributed solely to fucoxanthin but also due to other components of the algae, such as eicosapentaenoic acid (EPA) and fiber.

In another study involving human subjects, Hall et al. (2012) compared the energy intake of 12 healthy overweight and obese men at a test meal 4 h after taking bread enriched with the brown seaweed *Ascophyllum nodosum* (4% per 400 g wholemeal loaf) in a breakfast meal, with those taking the control bread (0% *Ascophyllum nodosum*) [22]. A significant reduction (16.4%) in energy intake was recorded amongst those given the seaweed-enriched bread in the subsequent 24 h after the test meal. The authors postulated that the reduced energy intake could be associated with the gastric stretching effects due to bulking resulting from the presence of alginates in the seaweed-enriched bread. However, there were no significant differences between the two treatment arms in terms of blood glucose and cholesterol levels.

Apart from using the whole seaweed, several studies have shown anti-obesity effects of seaweed extracts based on in vitro and in vivo models. For instance, Kang et al. (2016) assessed the anti-obesity effects of ethanol extracts from seaweeds collected from Jeju Island, Korea [23]. Of the extracts tested, the extract from the red seaweed *Plocamium telfairiae* was found to have the highest inhibitory effect on lipogenesis in adipocytes, especially in decreasing the expression of the adipogenic-specific proteins peroxisome proliferator-activated receptor-γ (PPAR-γ), cytosine-cytosine-adenosine-adenosine-thymidine (CCAAT)/enhancer-binding protein-α (C/EBPα), sterol regulatory element-binding protein 1 (SREBP 1), and fatty acid-binding protein 4. In another study, administration of extracts from the edible red seaweed *Gelidium amansii* caused body weight reduction in mice fed a high-fat diet [23]. The effect was attributed to suppressed adipogenic expression in adipocytes. Significant decrease in total cholesterol and triacylglycerol levels as well as blood glucose and insulin levels were also observed in the treated mice.

## 3. Marine Algae as a Source of Anti-Pancreatic Lipase Agents

Inhibition of lipases, especially pancreatic lipase, is one of the main therapeutic targets of anti-obesity drugs. The current approved anti-obesity drug in the market, orlistat, acts through this mechanism. Orlistat is a synthetic hydrogenated derivative of lipstatin, which acts as a potent, long acting reversible inhibitor of pancreatic and gastric lipases [6]. Lipstatin was first isolated from the actinobacterium *Streptomyces toxytricini*, and it contains a ß-lactone structure that probably accounts for the irreversible lipase inhibition [24]. The intense inhibitory activity of orlistat has raised concern as the absorption of some vitamins may also be inhibited [25]. Furthermore, orlistat treatment may result in the increased amount of fecal fat in the large intestine, and may promote colon carcinogenesis, as shown in animal study [26].

There have been intensive efforts to screen extracts from a wide range of natural sources, including plants, fungi, algae and bacteria for inhibitory activities against pancreatic lipase [27]. An extensive screening of methanol and ethyl acetate extracts from 54 species of marine algae for lipase inhibitors was conducted by Bitou et al. (1999) [28]. It was found that methanol extracts from *Caulerpa taxifolia* and *Asparagopsis tociformis* showed high activity (almost 100% inhibition) although similar extracts from other seaweeds such as *Codium latum*, *Gloiopeltis tenax*, *Hypnea charoides*, *Sargassum muticum* and *Dictyopteris latiuscula* were also promising. An active inhibitor, caulerpenyne was also isolated from the ethyl acetate extract of *Caulerpa taxifolia*. In addition, oral administration of corn oil with caulerpenyne demonstrated a reduced and delayed peak plasma triacylglycerol concentration in rats.

In another study, Rebah et al. (2008) showed that ethanol extracts from *Caulerpa prolifera* markedly reduced both dog gastric and human pancreatic lipase activities [29]. The authors further showed that fractionation of the crude extract by thin-layer chromatography (TLC) reduced the inhibitory rate, suggesting that the lipase inhibition may be caused by synergistic action of several compounds in the extract. A major compound with high lipase inhibition capacity was then isolated using HPLC in this study.

Recently, Chater et al. (2016) assessed the anti-pancreatic lipase activity of preparations from three brown seaweeds, namely *Ascophyllum nodosum*, *Fucus vesiculosus*, and *Pelvetia canaliculata* [30]. The preparations tested, which include the whole seaweed homogenate, sodium carbonate extract, and ethanol extracts (pellet and supernatant), showed significant inhibition of lipase activity. Multiple bioactive agents, including alginates, fucoidans and polyphenols of the extracts were suggested to be involved in exerting the inhibitory activity. The authors further validated the inhibitory effects of the extracts from *Fucus vesiculosus* using a model gut system. The supernatant fraction of the ethanol extract showed the strongest inhibition as indicated by the reduction in fat absorption.

Ethanol extracts, dried powders and fibers (total and soluble fibers) from the tropical edible red seaweeds *Kappaphycus striatus* and *Eucheuma denticulata* were assessed for their inhibitory activity against pancreatic lipase [31]. The ethanol extracts of dried *K. striatus* showed the highest inhibition (92%) against the enzyme followed by the seaweed powders, with average inhibition of 60%. However, the total dietary fiber extracts did not show significant inhibition against pancreatic lipase.

Fucoxanthin isolated from edible seaweeds, and its metabolite fucoxanthinol, have been shown to inhibit pancreatic lipase activity in the gastrointestinal lumen and leading to suppressed triacylglycerol absorption in lymph-duct cannulated rats [32]. In addition, fucoxanthin and fucoxanthinol were found to significantly inhibit pancreatic lipase activity, assayed based on inhibition of the hydrolysis of triolein. The inhibitory activities (IC_50_) of fucoxanthin and fucoxanthinol were similar but were much lower than orlistat.

Alginates are another group of seaweed compounds that have been shown to have inhibitory activity against pancreatic lipase. The inhibitory activity may vary with the source and chemical forms of the compound. For instance, Wilcox et al. (2014) reported that alginates from *Laminaria hyperborea,* with high glucoronic acid content, showed higher inhibition of pancreatic lipase than alginates from *Lessonia nigrescens,* with high mannuronic acid content [33]. The inhibition of pancreatic lipase by alginates is substrate specific, and is affected by the type of triacylglycerols and fatty acid chain length [34]. However, molecular weight of the alginates is not a determining factor that affects their inhibitory activity. The inhibitory activity of pancreatic lipase was not affected when alginates added to a bread vehicle were subjected to cooking at temperatures up to 200 °C or digestion in a model gut [35]. The mechanisms of action of alginates were suggested to involve interactions with the enzyme through hydrogen bonding by the hydroxyl group, charge–charge interactions with the carboxyl groups, and the negatively charged COO- group of the alginate. The technology of using alginates as an inhibitor of pancreatic lipase has been patented, and there is a potential application of the compound as an anti-obesity agent [36].

Phlorotannins are brown seaweed polyphenols known to prevent fat absorption by inhibiting pancreatic lipase. Of the various phloroglucinol derivatives, 7-phloroeckol is the most potent (IC_50_ = 12.7 μM) in inhibiting the enzyme activity although weaker than orlistat (IC_50_ = 0.7 μM) [37]. In comparison, the IC_50_ values of the other phloroglucinol derivatives, fucofuroeckol-A and eckol were 12 and 37 μM respectively. However, dieckol, dioxindehydroeckol and phlorofucofuroeckol did not seem to have any effect on pancreatic lipase activity.

## 4. Algal Compounds with Anti-Obesity Effects

There have been many reports on the potential anti-obesity agents derived from marine algae, particularly the seaweeds. Seaweed compounds such as alginates, fucoidans, and phlorotannins have been shown to have a role in the control of digestion and thus, have been implicated as potential agents for obesity treatments [34]. The carotenoid fucoxanthin is another potential agent with anti-obesity effect. Fucoxanthin is found in brown seaweeds as well as microalgae such as diatoms. The anti-obesity effects of the algal compounds, apart from their pancreatic lipase inhibitory activity, are covered in detail in the following sections. The mechanisms of action of the potential anti-obesity compounds from algae may involve alteration in lipid metabolism, suppression of inflammation, suppression of adipocyte differentiation and delay in gastric emptying (Table A1).

### 4.1. Fucoxanthin

Fucoxanthin is a marine carotenoid present mainly in brown seaweeds and diatoms, accounting for over 10% of total naturally produced carotenoids [38]. It is found abundantly in edible seaweeds such as *Undaria pinnatifida*, *Laminaria digitata* and *Hijikia fusiformis*. In addition, marine microalgae such as *Phaeodactylum tricornutum* and *Isochrysis galbana* have been regarded as potential commercial producers of fucoxanthin, as their contents of the pigment are much higher than those of brown seaweeds (Table A2) [39,40]. Fucoxanthin is a potent antioxidant compound, and has potential application for functional food preparation [41,42]. This carotenoid is also one of the most well-studied algal compounds in terms of anti-obesity effect [43,44]. The bioactivity of fucoxanthin is attributed to its polyene chromophore, which contains an allenic bond and two hydroxyl groups [44]. A summary of the various studies on the anti-obesity effects of fucoxanthin is given in Table A3.

Intake of fucoxanthin-rich Wakame has been shown to have anti-diabetic and anti-obesity effects in an obese mouse model [45]. There was suppression of weight gain and modulation of blood glucose and insulin levels in mice administered a high-fat diet followed by feeding of a fucoxanthin-rich diet. Feeding of KK-*Ay* mice with a combination of fucoxanthin with fish oil was found to reduce the gain of white adipose tissue (WAT) weight compared to those fed fucoxanthin alone [46]. The fucoxanthin-rich diet also suppressed MCP-1 mRNA expression in WAT, and thus, suppressed inflammatory reactions in those tissues. In another study, Tan and Hou (2014) demonstrated that feeding of fucoxanthin reduced the levels of inflammatory markers such as interleukin-1ß (IL-1β), TNF-α, inducible nitric oxide synthase (iNOS), and cyclooxygenase-2 (COX-2) in an obese mouse model [47]. Fucoxanthinol, a metabolite of fucoxanthin, was also reported to suppress low-grade inflammation in adipocytes, as indicated by its effect in reducing TNF-α and MCP-1 mRNA suppression in a co-culture of adipocytes and macrophage cells [48]. A more recent study by Grasa-Lopez et al. (2016) using a mouse model further proved that fucoxanthin decreased WAT mass, and decreased serum triacylglycerols but increased serum HDL cholesterol [21]. Other beneficial effects observed were improved insulin resistance, reduced blood pressure, increased expression and decreased serum levels of adiponectin, and decreased expression of leptin. There was also enhanced β-oxidation resulting from the increased expression of uncoupling protein 1 (UCP-1).

Several studies have shown that fucoxanthin ameliorates obesity through its effects on lipid metabolism. For instance, feeding of seaweed extract containing fucoxanthin was found to enhance β-oxidation and reduce lipogenesis in an obese mouse model [49]. In addition, fucoxanthin increased the activities of key enzymes in lipid metabolism, namely AMP-activated protein kinase (AMPK) and acetyl-CoA carboxylase (ACC) in epididymal adipose tissue. A combination of fucoxanthin and conjugated linoleic acid has been shown to reduce serum levels of triacylglycerols, glucose and leptin in diet-induced obese rats [50]. Conjugated linoleic acid is a group of isomers of linoleic acid, of which the *trans-*10, *cis-*12 isomer is known to have an anti-obesity effect. The WAT weight gain decreased in rats fed fucoxanthin and conjugated linoleic acid, but not in those fed fucoxanthin alone. The combination was also found to regulate mRNA expression of enzymes related to lipid metabolism (e.g., carnitine palmitoyltransferase 1A) in WAT of the rats. Fucoxanthin also exerts its effect on hepatic lipid contents by regulating metabolic enzyme activities and stimulating fatty oxidation activity [51]. Such effect was due to the reduced activity of the hepatic lipogenic enzymes, glucose-6-phosphate dehydrogenase, malic enzyme, fatty acid synthase and phosphatidate phosphohydrolase, and the enhanced activity of β-oxidation, as demonstrated in a mouse model. The anti-obesity effect of fucoxanthin is also due to its induction of UCP1 expression in WAT, which enhances the dissipation of energy through oxidation of fatty acids and heat production [52]. For instance, in mice fed with capsules containing fucoxanthin-rich Wakame lipids, increased levels of UCP1 and mRNA expression of UCP1 were observed [53].

Dietary fucoxanthin is converted to fucoxanthinol in the gastrointestinal tract before absorption in the intestine, and this is subsequently converted to amarouciaxanthin A in the liver of mice [54]. The percentage accumulations of fucoxanthin, fucoxanthinol, and amarouciaxanthin in the adipose tissue of mice were 13%, 32% and 55%, respectively [55]. The metabolite fucoxanthinol was found to downregulate PPARγ and exhibit stronger suppressive effects on adipocyte differentiation compared to fucoxanthin [56]. The accumulatation of fucoxanthinol in WAT suggests that dietary fucoxanthin could be a useful natural compound for the prevention of obesity.

Fucoxanthin has also been shown to affect the differentiation of adipose-derived stem cells (ADSC), reducing the elevation of reactive oxygen species (ROS) and lipid droplet accumulation in those cells induced by palmitic acid [57]. In addition, fucoxanthin reversed the decrease of mRNA levels of lipid metabolism genes such as *Leptin*, *Adiponectin, ATGL* and *SIRT1* in the ADSC induced by palmitic acid. Fucoxanthin showed varying effects on the three differentiation stages of 3T3-L1 preadipocytes [58]. During early differentiation stages (days 0–2, D0–D2), fucoxanthin promoted adipocyte differentiation and increased protein expression of PPARγ, CCAAT/C/EBPα, SREBP1c and adiponectin mRNA expression. However, fucoxanthin reduced the expression of PPARγ, C/EBPα, and SREBP1c during the intermediate (D2–D4) and late stages (D4–D7) of differentiation. In addition, it inhibited the uptake of glucose in mature 3T3-L1 adipocytes by reducing the phosphorylation of insulin receptor substrate 1 (IRS-1).

In view of the potential use of fucoxanthin as a nutraceutical, especially for its anti-obesity activity, several toxicity studies have been conducted as part of its safety evaluation. In one study, rats fed repeated oral doses of fucoxanthin (95% purity) for 4 weeks did not show any significant toxicity [59]. Another study showed that there was no acute toxicity in rats fed with extracts containing 0.0012% fucoxanthin after a 4-week daily treatment [60]. In another study, both single (1000 and 2000 mg/kg body weight) and repeated dose (500 and 1000 mg/kg body weight, 30 days) toxicity testing of fucoxanthin in mice showed that there were no mortalities or abnormalities in gross appearance [61]. However, significant increase in plasma total cholesterol was shown in mice fed with fucoxanthin.

Commercial formulation of fucoxanthin as a nutraceutical with anti-obesity activity is already available in the market. The commercial product Xanthigen contains a combination of fucoxanthin and punicic acid, a fatty acid found in pomegranate oil [62]. The product was tested in a clinical trial involving obese and non-diabetic female volunteers with NFLD and normal liver fat. Results showed that the supplement reduced body and liver fat content, and improved liver function tests in those subjects. Xanthigen was also shown to suppress adipocyte differentiation and lipid accumulation through multiple mechanisms [63]. The effects include down-regulation of the protein levels of key transcription factors (e.g., PPARγ) and enzymes (e.g., fatty acid synthase) in adipogenesis. A recent intervention study on two healthy premenopausal obese women showed that consumption of Xanthigen induced expression of brown adipose tissue (BAT) although there was no weight reduction [64]. Accumulation of BAT is desirable as it is associated with energy expenditure, in contrast to WAT, which is involved in fat storage. The expression of BAT was detected in both cervical, supraclavicular and paravertebral spaces in one subject, as assessed by 18F-fluorodeoxyglucose (FDG) positron emission tomography (PET). However, as suggested by the authors, a further large well-controlled study is needed to confirm the findings.

### 4.2. Alginates

The potential health benefits of seaweeds are partly due to their high contents of dietary fibers, which are not digested to any great extent in the gut [16]. Such fibers can increase satiety feeling and aid digestive transit through their bulking capacity. Alginates are amongst the seaweed fibers that are well-known for their anti-obesity effects. Alginates include salts and derivatives of alginic acid, a gelling polysaccharide that forms the structural component of brown seaweeds [65]. Alginates comprise up to 40% of the dry weight of brown seaweeds [66]. Most of the commercially used alginates are sourced from brown seaweeds of the genera *Laminaria*, *Macrocystis* and *Ascophyllum* and *Lessonia* [67]. *Laminaria* and *Lessonia* species are the dominant source of alginates, representing 81% of the seaweed harvests. Alginates are widely used in the food industry as a thickener or emulsification stabilizer in food products. Sodium alginate is the most common form of alginates used in the food industry.

Dietary alginate supplementation has been shown to increase satiety, reduce energy intake and support weight reduction [12]. For instance, consumption of a post-ingestion, calcium-gelled fiber beverage twice daily reduced energy intake in overweight and obese women with low rigid restraint scores [68]. Mechanisms involved in exerting the effect include delayed gastric clearance, stimulation of gastric stretch receptors and attenuated nutrient absorption. Most studies on alginates were conducted using commercial formulations of alginate drinks. In one study, subjects were given chocolate milk with 2.5% alginate and it was found that this reduced mean appetite by 134% compared with chocolate milk alone [69].

While alginate consumption has been shown to affect satiety feelings and energy intake, most studies have been of short duration rather than assessing the long-term effects [12]. Arshad et al. (2016) compared the postprandial glycemic and satiety responses of different dietary polysaccharides (carrageenan, guar gum and alginates) when added in milk (2% Milk Fat) in 30 females [70]. Following an ad libitum pizza meal, both alginates and guar gum suppressed glucose and average appetite of the subjects although the effect of guar gum was more pronounced. In addition, administration of alginates resulted in lower blood glucose levels compared with the control and carrageenan during post-treatment.

Satiety effect is an important factor that regulates food intake and thus, it has great significance in the control of obesity. Physical properties such as viscosity and gel strength may also influence the satiety effect of alginates. For instance, Solah et al. (2010) showed that an alginate-based drink with high viscosity had a more pronounced effect in reducing hunger of the subjects compared to that with low viscosity [71]. The study also showed that viscosity reduced hunger more than the protein effect in subjects given alginate-based drinks with different viscosities and protein contents. Another study by Hoad et al. (2009) showed that gastric emptying was delayed in the presence of strongly gelled alginate beads compared to weakly gelled beads in human subjects [72].

The anti-obesity effect of alginates may be influenced by the conformational structure of the polysaccharide. For instance, Nakazono et al. (2016) compared the anti-obesity effects of dietary acid-hydrolyzed (A-AO) and enzymatic-digested (E-AO) alginate oligomers in mice fed a high-fat diet [73]. The anti-obesity effects of E-AO were stronger than A-AO in terms of reduction in body and adipose tissue weights. In addition, in vitro studies showed that E-AO, but not the full polymer, inhibited lipid accumulation in differentiated 3T3-L1 adipocytes.

An extensive review on the effects of alginate supplementation on appetite regulation, glycemic and insulinemic responses, and lipid metabolism in animal and human studies was published by Georg Jensen et al. (2013) [74]. The efficacy could be influenced by the dose, ß-1,4-d-mannuronic acid (M):α-1,4-l-guluronic acid (G) (M:G) ratio and viscosity of the alginates administered. Most of the animal studies showed that alginate consumption resulted in suppressive effect on food intake and reduction in body weight. Some of the proposed mechanisms for the effects include gastric distension, impaired nutrient digestibility, and absorption related either to gel formation or the increased viscosity of the gastric content. The review also concluded that, in terms of acute effect, alginate supplementation suppresses appetite feelings compared to the control treatments. As for short-term effect, there is evidence showing that the form of alginate administered does affect the outcome of the reported intervention studies. Alginate supplementation in hydrated form, using a beverage as a vehicle, does suppress food intake. However, when alginates are incorporated into solid food or capsules in combination with other dietary fibers, they do not seem to have a significant effect on appetite or energy intake in free-living conditions. As stressed by Lange et al. (2015), due to the limited long-term intervention studies, it may not be possible to critically assess the potential of alginate supplementation in improving body weight regulation [12].

Animal and human studies have also shown that alginate consumption exhibits beneficial effects on postprandial glucose absorption and insulin response [74]. The effect has been suggested to be due to the gelling properties and viscosity of alginates, similar to other dietary fibers such as ß-glucans and pectin [75]. In addition, administration of calcium-alginate has been shown to reduce blood cholesterol levels in rats fed a high cholesterol diet [76]. The proposed mechanism is that enhanced fecal excretion of bile acid due to reduced intestinal reabsorption could stimulate bile acid synthesis from cholesterol in the liver, resulting in a decrease in plasma cholesterol. Sodium alginate has also been shown to reduce the permeability of intestinal mucus, which implicates that this may reduce problems associated with high rates of lipid absorption such as hyperlipidemia [77].

### 4.3. Fucoidans

Fucoidans are a type of highly sulfated polysaccharides, consisting of high amounts of fucose, found mainly in the extracellular matrix of brown seaweeds [78]. These polysaccharides also contain one or more small proportions of d-xylose, d-mannose, d-galactose, l-rhamnose, arabinose, glucose, d-glucoronic acid and acetyl groups, which vary amongst different seaweed types. Most reports on the bioactivity of fucoidans are related to anti-tumor activity [78]. However, there are also studies which demonstrate that fucoidans do have anti-obesity effects. For instance, fucoidans extracted from *Undaria pinnatifida* have been shown to have anti-adipogenic activity by down-regulating mRNA gene expression of key adipogenic markers (e.g., adipocyte protein 2) and expression of inflammation-related genes in adipocytes during adipogenesis. Furthermore, fucoidans decreased the accumulation of lipids and ROS in adipocytes. Fucoidans were also found to inhibit lipid accumulation in differentiated 3T3-L1 adipocytes [79]. The expression of hormone-sensitive lipase, the key enzyme involved in lipolysis, was also increased. The findings suggest that fucoidans reduce lipid accumulation by stimulating lipolysis. The same study also showed that insulin-induced 2-deoxy-d-[3H] glucose uptake was decreased up to 51% in fucoidans-treated cells as compared to the control. In another study, Kim et al. (2009) also demonstrated that fucoidans inhibited adipogenesis by suppressing the expression of genes coding for fatty acid binding proteins (aP2), acetyl CoA carboxylase (ACC) and peroxisome PPARγ [80]. Fucoidans were also found to suppress expression of inflammation-related cytokines such as TNFα, MCP-1 and plasminogen activator inhibitor-1 (PAI-1) in 3T3-L1 adipocytes [81].

The anti-obesity effects of fucoidans have also been demonstrated in animal model studies. For instance, Kim et al. (2014) demonstrated that high-fat diet mice fed with fucoidans showed a decrease in body-weight gain, food-efficiency ratio and relative liver and epididymal fat mass compared to those on high-fat diet alone [82]. There was also a decrease in the plasma levels of triacylglycerols, total cholesterol and low-density lipoprotein (LDL) in the mice fed with fucoidans. In addition, there was down-regulation of the epididymal adipose genes coding for aP2, ACC and PPARγ. In another study, administration of fucoidans extracted from *Sargassum henslowianum* were found to lower blood cholesterol, triacylglycerol and LDL-cholesterol levels in obese mice [83].

There have been limited studies on fucoidans in human subjects. A randomized, double-blind, placebo-controlled clinical trial on fucoidans was conducted on 25 obese or overweight human subjects [84]. Fucoidan administration during the three-month period resulted in decreased diastolic blood pressure and LDL-cholesterol levels, increased insulin secretion and insulin resistance in the overweight or obese adults.

### 4.4. Phlorotannins

Phlorotannins are phloroglucinol-based polyphenols commonly found in brown seaweeds. They are synthesized through the polyketide pathway, and consist of phloroglucinol monomers with molecular weights ranging from 126 to 650 kD [85]. The phloroglucinol units may be linked up in various ways, giving a great variety of the compounds. Phlorotannins can be classified into four subclasses, based on the type of linkage, namely fuhalols and phlorethols (phlorotannins with an ether linkage), fucols (with a phenyl linkage), fucophloroethols (with an ether and phenyl linkage), and eckols (with a dibenzodioxin linkage) [86]. Some of the pholorotannins are halogenated, containing bromine, chlorine or iodine. The compounds are thought to be produced by seaweeds to cope with stressed conditions and to defend against herbivores [85]. Brown seaweeds which are known to produce bioactive pholorotannins include *Eisenia bicyclis*, *Ecklonia cava*, *Ecklonia stolonifera*, *Undaria pinnatifida*, *Sargassum thunbergii*, *Ishigeo kamurae* and *Laminaria japonica* [87]. Of the brown seaweeds, *E. cava* contains the highest amount of phlorotannins [11].

Phlorotannins alleviate obesity and obesity-related disorders through several mechanisms, including inhibition of pancreatic lipase (see Section 3) and obstruction of adipocyte differentiation. For instance, phlorotannins from *E. cava* and *E. stolonifera* suppressed lipid accumulation during adipogenesis without affecting cell viability [88]. Phloroglucinol and eckol suppressed adipogenesis by down-regulating C/EBPα and PPARγ while dieckol acted by activating AMP activated protein kinase (AMPK) and inhibiting PPARγ [89]. Another phlorotannin, diphlorethohydroxycarmalol (DPHC), from *Ishigeo kamurae,* caused apoptosis of preadipocytes [90]. However, there have been no reports on studies on mature adipocytes.

The anti-adipogenesis effect of phlorotannins could be due to their effect in inhibiting the peptidyl prolyl *cis/trans* isomerase Pin1 [91]. Pin1 is involved in enhancing the uptake of triacylglycerols and the differentiation of fibroblasts into adipocytes in response to insulin stimulation. Recently, Han et al. (2016) showed that Pin1 regulates adipogenesis by regulating the transcriptional activity of PPARγ [92]. Down regulation of Pin1 could be a potential therapeutic target for obesity-related disorders. A phlorotannin with Pin1 inhibitory activity has been isolated from the seaweed *Ecklonia kurome* [91].

Another effect of phlorotannins is the inhibition of protein tyrosine phosphatase 1B (PTP1B). As PTP1B is involved in negatively regulating insulin signal transduction, its inhibition is an attractive target for treating obesity [93]. Eckol, phlorofucofuroeckol-A, dieckol and 7-phloroeckol are potent and non-competitive PTP1B inhibitors, with IC_50_ (0.56 to 2.64 μM) much lower than the positive control of ursolic acid (10.82 μM) [94].

Of the phlorotannins, eckol appears to be the most promising target compound for anti-obesity drug development due to its multiple inhibitions on pancreatic lipase, adipogenesis and PTP1B [11]. However, most of the studies on phlorotannins were in vitro (cell lines and enzyme inhibitory assay). Further studies using animal model and human subjects are worthwhile to assess the efficacy of phlorotannins as anti-obesity agents.

## 5. Future Directions of Research

While the bioactive compounds from algae mentioned above show promising potential as anti-obesity agents, factors such as their stability and bioavailability need to be considered. There is also a need to develop effective delivery systems for the algal compounds. For instance, the main obstacle in using alginates for obesity treatment appears to be how to introduce the fiber into the everyday diet [34]. While alginates have been added to food and drinks (e.g., white bread), the alginate-enriched products may have poor palatability. In addition, new delivery systems for fucoxanthin need to be further explored as the bioavailability of the carotenoid has been shown to be low in human subjects [95]. Research by Salvia-Trujillo et al. (2015) assessed the bioavailability of fucoxanthin from nanoemulsion-based delivery systems using different lipid carrier types [96]. Absorption rate of fucoxanthin into intestinal epithelial cells from the nanoemulsion using long-chain triacylglycerols carrier oil was found to be higher than that based on medium-chain triacylglycerols (MCT). Other groups have developed a biodegradable chitosan-glycolipid hybrid nanogel system to encapsulate fucoxanthin to improve its stability and bioavailability [97].

As digestion and absorption of dietary lipids are a major source of excess calories, they are the primary targets for development of anti-obesity drugs [98]. As such, finding new compounds with pancreatic lipase inhibitory activity will be the way forward. Although there is much research elucidating potential pancreatic lipase inhibitors, very few compounds have entered clinical trials, and no new drug with such activity has been marketed after orlistat [98]. Algal compounds with inhibitory activity against pancreatic lipase could be useful as anti-obesity agents. However, most of the studies on anti-pancreatic lipase activity have been based on crude extracts [30,31] although algal compounds such as alginates, fucoxanthin and phlorotannins have been shown to have potent lipase inhibitory activity [34,99]. Further studies on detailed structure–activity relationship on semi-synthetic and synthetic derivatives of these algal compounds are needed if potential leads are to be developed for the treatment and prevention of obesity [34].

Bioactive compounds that inhibit gastric and pancreatic lipase activity appear to be the main target in the search for anti-obesity agents. However, molecules that affect enzymes involved in other stages of lipid metabolism should also be targeted for new pharmacotherapies [100]. Such targets include enzymes and proteins that are involved in lipid transport and absorption, such as pancreatic phospholipase A2 (sPLA2), fatty acid-transport protein-4 (FATP4), monoacylglycerol acyltransferase (MGAT) and microsomal triglyceride-transfer protein (MTP) [100]. The testing of potential anti-obesity agents should be on multiple target platforms instead of a single molecular target.

The area that has attracted much interest for potential future weight loss strategies is the selective modulation of the intestinal microflora [8]. Comparison of the gut microbiota of obese and lean human volunteers has shown that obesity is associated with changes in the relative abundance of the two dominant bacterial divisions, the *Bacteroides* and the *Firmicutes* [101]. Metagenomic and biochemical analyses have also revealed that the obese microbiome has an increased capacity to harvest energy from the diet, resulting in a significantly greater increase in total body fat than colonisation with a “lean microbiota”. It would be interesting to investigate how algal compounds such as alginates, fucoxanthin and phlorotannins affect the gut microbiome in obese and normal individuals. Previous studies have shown that consumption of alginates could alter the colonic microflora, depending on the time and levels of alginate exposure [102]. For instance, there was a report on the effects of alginate consumption (10 g/day over a 2-week period feeding versus alginate-free control diet) on human fecal bacteria [103]. It was found that fecal bifidobacteria levels increased, while the number of bacteria such as Enterobacteriaceae decreased during alginate consumption.

Weight gain could be a side effect of certain adipogenic medications such as antipsychotic and antidepressant drugs. For instance, antipsychotics such as clozapine and olanzapine have adipogenic potential, especially in influencing adiponectin and leptin levels, and carry the risk for the development of hypertension, diabetes and lipid abnormalities [104]. The potential application of fucoxanthin as a supplement when drugs with known weight gain effects are prescribed, are worth exploring [43].

Development of bioactives from marine algae as therapeutic agents (pharmaceutical drugs) for obesity may need more intensive and longer studies, especially human trials. While there have been many studies on the anti-obesity effect of alginates, most have been short duration, which are not able to assess long-terms effects on appetite and energy intake [12]. From another perspective, it is worth exploring the potential applications of the algal compounds as ingredients in functional food or as dietary supplements. Such bioactive compounds may have low competency compared to pharmaceutical drugs. However, when taken as functional food, the bioactives are taken regularly as part of the diet, and may have a noticeable long-term physiological effect and beneficial in terms of long-term weight management [105]. Finally, it also needs to be established whether anti-obesity effects of seaweed consumption vary in different human populations [12].

## 6. Conclusions

Marine algae, particularly seaweeds as a dietary component, are beneficial in the management of body weight and obesity. Algal compounds such as fucoxanthin, alginates, fucoidans and phlorotannins have potential application as anti-obesity agents. Such compounds could be useful as nutraceuticals or dietary supplements for body weight and obesity management. However, there is still a need for more human trials of longer duration to assess the efficacy of such compounds as anti-obesity agents. For the development of new anti-obesity drugs, especially pancreatic lipase inhibitors, further studies on structure–activity relationships are required to establish new pharmacological leads based on the algal products.

## Appendix A

**Table A1:** Mechanisms of action of potential anti-obesity compounds from marine algae.

Algal Compounds	Mechanism of Action	Reference
Fucoxanthin	Inhibition of pancreatic lipase	[32]
Alginates		[33]
Phlorotannins		[37]
Fucoxanthin	Enhanced ß-oxidation through increased expression of uncoupling protein 1	[21]
	(UCP-1)	
	Suppression of inflammation in white adipose tissues (WAT)	[45]
	Increased activities of key enzymes in lipid metabolism—AMP-activated protein kinase (AMPK) & acetyl CoA carboxylase	[49]
Fucoxanthin	Suppression of adipocyte differentiation	[56]
Phlorotannins		[88]
Alginates	Delayed gastric clearance, stimulation of gastric stretch receptors and attenuated nutrient absorption	[68]
Fucoidans	Downregulation of gene expression of key adipogenic markers and inflammatory-related genes in adipocytes	[78]

**Table A2:** Fucoxanthin contents in marine algae, expressed on the basis of algal weight.

Algal Producer	Content	Remarks	Reference
**Microalgae**			
*Phaeodactylum tricornutun*	15.42–16.51 mg/g freeze-dried sample weight	Ethanol provided the best extraction yield; The diatom also contained high amounts of EPA	[39]
*Isochrysis galbana*	18.23 mg/g dried sample	Most fucoxanthin (~95%) could be extracted in ethanol	[40]
*Chaetoceros calcitrans*	5.25 mg/g dry weight	Preparation of a fucoxanthin-rich fraction with high antioxidative properties	[106]
*Odontella aurita*	18.47 mg/g dry weight	Grown in a bubble column photobioreactor; low light and nitrogen-replete culture medium enhanced biosynthesis of fucoxanthin	[107]
**Seaweeds**			
*Sargassum horneri* (Akamoku)	10.81 mg/g dry weight	The contents varied with season—highest in samples harvested during the coldest part of the year	[108]
*Laminaria japonica* (konbu)	0.19 mg/g fresh weight	Extracted from waste parts of the cultured seaweed	[109]
*Laminaria japonica*	0.03 mg/g fresh weight	Microwave-assisted extraction coupled with high-speed countercurrent chromatography	[110]
*Undaria pinnatifida* (wakame)	0.73 mg/g dry weight		
*Sargassum fusiforme*	0.01 mg/g dry weight		
*Petalonia binghamiae*	0.43–0.58 mg/g fresh weight	Deep seawater was used for the culturing of the seaweed	[91]

**Table A3:** Summary of findings from the literature on the anti-obesity activities of fucoxanthin.

Test Material/Chemical	Experimental Model	Findings	Reference
Xanthigen (brown marine algae fucoxanthin + pomegranate seed oil)	Mouse 3T3-L1 preadipocytes	↓ accumulation of lipid droplets in adipocytes; ↓ protein levels of key adipogenesis transcription factors: PPARγ, CCAAT/enhancer binding protein (C/EBP) β, and C/EBPδ & fatty acid synthase; ↑ NAD^+^-dependent histone deacetylases (SIRT1) and activated AMP-activated protein kinase (AMPK) signaling in differentiated 3T3-L1 adipocytes.	[63]
Brown seaweed extract (10% fucoxanthin)	Human adipose-derived stem cells	↓ ROS; Silencing of palmitic acid-induced long non-coding RNAs (lncRNAs) resulted in the decrease in lipid droplet accumulation	[57]
Fucoxanthin-rich wakame (*Undaria*) lipids (WL)	Mouse (Type 2 diabetes/obese model)	↓ body weight and white adipose weight; ↓ plasma levels of leptin; ↑ mRNA expression of β3-adrenergic receptor (Adrb3) in WAT and glucose transporter 4 (GLUT4) mRNA in skeletal muscle tissues	[45]
Fucoxanthin & Fucoxanthinol	Mouse (Type 2 diabetes/obese model)	Improved glucose tolerance; ↓ TNF-γ and MCP-1 expression in WAT	[48]
Capsule containing omega-3 PUFA-rich scallop phospholipids (PL) with incorporation of *Undaria* lipids (UL) containing fucoxanthin	Mouse	↓ body weight and WAT weight; ↑ UCP1 and mRNA expression of UCP1 in epididymal fat	[53]
Fucoxanthin (pure chemical)	Mouse	↓ IL-1β, TNF-α, iNOS, and COX-2, and suppressed maleic dialdehyde (MDA) and infiltration of polymorphonuclear cells (PMN)	[47]
*Petalonia binghamiae* extract containing fucoxanthin	Mouse	↓ body weight gain, adipose tissue weight and cell size, fatty droplet accumulation in the liver, and serum triacylglycerol level; ↓ phosphorylation of AMP-activated protein kinase (AMPK) and acetyl-CoA carboxylase (ACC) in epididymal adipose tissue	[58]
Fucoxanthin (isolated from dried *Undaria pinnatifida*)	Mouse	↓ plasma triacylglycerols with a concomitant; ↑ fecal lipids; ↓ hepatic lipid contents; ↓ activity of the hepatic lipogenic enzymes, glucose-6-phosphate dehydrogenase, malic enzyme, fatty acid synthase and phosphatidate phosphohydrolase; ↑ activity of β-oxidation; ↑ plasma HDL-cholesterol concentrations; ↓ glucose and HbA1c	[51]
Fucoxanthin oil (1% fucoxanthin) + conjugated linolenic acid (CLA)	Rat	↓ triacylglycerol and leptin levels; ↑ mRNA expression of adiponectin, adipose triacylglycerol lipase and carnitine palmitoyltransferase 1A	[50]
Xanthigen (brown marine algae fucoxanthin + pomegranate seed oil)	Human subjects	↓ body weight, waist circumference, body and liver fat content, liver enzymes (NAFLD group only), serum triacylglycerols and C-reactive protein	[62]
